# Clones of Ectopic Stem Cells in the Regeneration of Muscle Defects *In Vivo*

**DOI:** 10.1371/journal.pone.0013547

**Published:** 2010-10-20

**Authors:** Rujing Yang, Mo Chen, Chang Hun Lee, Richard Yoon, Shan Lal, Jeremy J. Mao

**Affiliations:** 1 Tissue Engineering and Regenerative Medicine Laboratory (TERML), Department of Growth and Development, Columbia University Medical Center, New York, New York, United States of America; 2 Department of Biomedical Engineering, Columbia University, New York, New York, United States of America; University of Crete, Greece

## Abstract

Little is known about whether clones of ectopic, non-muscle stem cells contribute to muscle regeneration. Stem/progenitor cells that are isolated for experimental research or therapeutics are typically heterogeneous. Non-myogenic lineages in a heterogeneous population conceptually may compromise tissue repair. In this study, we discovered that clones of mononucleated stem cells of human tooth pulp fused into multinucleated myotubes that robustly expressed myosin heavy chain *in vitro* with or without co-culture with mouse skeletal myoblasts (C2C12 cells). Cloned cells were sustainably Oct4+, Nanog+ and Stro1+. The fusion indices of myogenic clones were approximately 16–17 folds greater than their parent, heterogeneous stem cells. Upon infusion into cardio-toxin induced tibialis anterior muscle defects, undifferentiated clonal progenies not only engrafted and colonized host muscle, but also expressed human dystrophin and myosin heavy chain more efficaciously than their parent heterogeneous stem cell populations. Strikingly, clonal progenies yielded ∼9 times more human myosin heavy chain mRNA in regenerating muscles than those infused with their parent, heterogeneous stem cells. The number of human dystrophin positive cells in regenerating muscles infused with clonal progenies was more than ∼3 times greater than muscles infused with heterogeneous stem cells from which clonal progenies were derived. These findings suggest the therapeutic potential of ectopic myogenic clones in muscle regeneration.

## Introduction

Much knowledge has been gained on the capacity of muscle derived stem/progenitor cells in muscle repair [1]–[3]. However, autologous muscle-derived cells in muscular dystrophy patients can be scarce as a therapeutic cell source. Ectopic, non-muscle derived stem/progenitor cells may act as adjunctive or alternative cell sources for muscle regeneration. Bone marrow-derived stem/progenitor cells fuse with degenerated muscle fibers in mdx mice, and participate in muscle regeneration [4]. MyoD positive, adipose-derived stem cells merge with native myoblasts in mdx mice *in vitro*, and restore dystrophin expression *in vivo* [5]. Human fetal blood cells differentiate into myogenic cells, and upon continuous exposure to galectin-1, engraft into toxin-induced or mdx muscles [6]. Pericytes isolated from blood vessels of human skeletal muscle engraft in mdx mice and express human dystrophin [7]. Intramuscular or intra-arterial injections of dental pulp cells into muscular dystrophy dogs lead to sparse engraftment and faint dystrophin expression, despite the infusion of a large number of cells (6×10^7^/mL) [8]. However, most ectopic stem cells previously used for muscle healing have been heterogeneous. Non-myogenic cells of the heterogeneous population conceptually may compromise the efficacy of muscle repair.

The suboptimal efficacy of heterogeneous stem cells in muscle regeneration has prompted recent interest in exploring single cell clones. A single muscle stem cell depleted of endogenous satellite cells infused into the tibialis anterior muscles of mice is capable of substantial self-renewal and differentiation *in vivo* [9]. Single cell clones isolated from mdx mice express several characteristic markers and show multipotency, and engraft with host muscle upon *in vivo* transplantation [10]. However, a direct comparison of the efficacy of heterogeneous stem cells and their clonal progenies has yet to be made. Here, we discovered that several clones of ectopic, heterogeneous stem/progenitor cell populations isolated from human tooth pulp were particularly prone to myogenic differentiation. These clones fused into multinucleated cells *ex vivo* and robustly expressed myosin heavy chain (MHC) in either chemically defined medium or upon co-culture with mouse skeletal myoblasts (C2C12 cells). We then infused a Nanog+, Oct4+, Stro1+, but CD133- and CD146- clone into cardiotoxin-injured tibialis anterior (TA) muscles in NOD/SCID mice. This ectopic, undifferentiated clone not only engrafted, but also expressed human-specific dystrophin and MHC, more efficaciously than their parent heterogeneous cells. Together, myogenic clones of heterogeneous stem cells may have value in muscle regeneration.

## Materials and Methods

### Ethics Statement

All research involving human participants was approved by Columbia University Medical Center Institutional Review Board (IRB). Dental stem/progenitor cells (DSCs) were isolated from infection-free deciduous teeth of 6 donors (5–9 yrs old) following IRB approval and informed parent consent. Consent statement was verbal because the study materials were regarded as medical waste.

### Cell isolation

Tooth pulp was minced and digested with collagenase (3 mg/mL) and dispase (4 mg/mL) for 1 h at 37°C. Mononucleated and adherent cells were isolated by single cell suspension and passage of the cells through a 70-µm strainer (BD, Franklin Lakes, NJ). The isolated cells were cultured in Dulbecco's Modified Eagle Medium: Nutrient Mixture F-12 (DMEM/F12, 1∶1, Invitrogen, Carlsbad, CA) supplemented with 10% Fetal bovine serum (FBS, Atlanta Biological, Lawrenceville, GA) and 1% Antibiotic-Antimycotic (Atlanta Biologicals) at 37°C and 5% CO_2_ with medium change twice a week. The growing culture was maintained for 2 wks, and then washed twice in PBS, dissociated in 0.25% Trypsin-EDTA (Mediatech, Manassas, VA).

### Cloning

A total of 42 clones were established by limiting dilution from heterogeneous DSCs. Passage 0 heterogeneous cells were seeded in 96-well plates at a concentration of 3 cells/mL with 200 µL medium per well. After 24 hrs, each of the wells with only one cell was selected for clonal expansion at 37°C and 5% CO_2_. After 3–4 wks, single-cell derived clones were treated with 0.25% trypsin-EDTA and seeded into 24-well plates for further expansion. Clonal progeny was transferred to 6-well plates upon ∼80% confluence, and at 37°C and 5% CO_2_ with medium change twice a week. Clonal progeny was passaged for the tested ∼20 doublings. Passage-5 clonal progeny cells were used for *in vivo* cell delivery.

### Self renewal and myogenic differentiation

Heterogeneous cells and clonal progenies were separately plated at a density of 5×10^3^ cells per cm^2^, and passaged. At each passaging, cell numbers were counted using a haemocytometer. The doubling time was calculated as the number of divisions = log_2_ (number of cells at subculture/number of cell seeded) [11]. At passage 2, 5, and 17 for heterogeneous DSCs and P5 and P9 for clonal progenies, CD34, CD44, CD105, CD133, CD146, stro1, Oct4 and Nanog were assayed by immunefluorescent antibodies and cell counting in at least 10 representative fields. At least 600 cells were counted per cell surface or nuclear marker in triplicates (Table 1). Subpopulations of heterogeneous cells and clonal progenies were plated at a density of 3,000 cells/cm^2^ in 24 well plates and exposed to myogenic differentiation medium, consisting of 97% DMEM, 2% horse serum and 1% Antibiotic-Antimycotic. Myogenic clones were confirmed by immunocytochemistry and real-time PCR for the expression of myosin heavy chain (MHC), and the formation of multi-nucleated myotube-like structures. Myogenic clones were distinguished from other clones of the same ectopic stem/progenitor cell population: myogenic clones readily transformed into MHC-positive myotube-like structures, whereas non-myogenic clones failed to express MHC or form myotube-like structures. In parallel with myogenic differentiation in chemically defined medium, we also attempted to induce myogenic differentiation by co-culture of heterogeneous cells and clones (2500/cm^2^) separately with mouse skeletal myoblasts (C2C12 cells) (2500/cm^2^) for 3 days in DMEM (high glucose) supplemented with 10% FBS and 1% Antibiotic-Antimycotic. Then, the co-cultured cells were treated with myogenic differentiation medium consisting of 97% DMEM (high glucose) supplemented with 2% horse serum and 1% Antibiotic-Antimycotic for 5 additional days. The cells were then fixed in 10% formalin and stained with anti-human nuclei and anti-MHC antibodies.

**Table 1:** Molecular markers of the native, heterogeneous dental stem/progenitor cell population and two myogenic clones, B6 and C3.

	DSCs			B6		C3	
	P2	P5	P17	P5	P9	P5	P9
**CD133**	39/642	0/623	0/904	0/813	0/672	0/627	0/627
**CD146**	229/606	114/702	0/782	0/813	0/672	0/627	0/627
**Stro1**	67/689	27/629	1/832	620/684	639/703	683/714	586/637
**Oct4**	572/603	590/630	-	692/710	520/622	642/724	411/710
**Nanog**	580/624	623/671	-	518/629	592/632	556/659	361/673

Frequency of positive marker expression expressed as the number of positive cells among total counted cells. P: cell passage. “-”: absent expression.

### *In vivo* cell engraftment

#### Ethics Statement

All animal work was approved by the IACUC and conducted according to relevant national and international guidelines. The work further adhered to NIH Animal Research guidelines. Following IACUC approval, NOD/SCID mice (8–10-wk-old) were anesthetized with 1–3% Isoflurane, followed by multi-point injection of Cardiotoxin I from Naja naja atra (Sigma, C3987, 0.5 µg in 30 µl PBS per muscle) in the right TA muscle (N = 10). The left TA muscle was injected with PBS (same volume) and served as control. Twenty-four hrs following cardiotoxin injections, human cells (1×10^6^) were multi-point infused into the injured TA muscles. Mice were sacrificed 4 wks later, followed by careful dissection of the TA muscles and embedding using OCT mounting matrix. The blocks were sectioned at 7-µm thickness using a cryostat. Human cell incorporation was assessed as the number of human dystrophin-positive myofibers divided by total myofibers per section using serial sections at 100-µm intervals along the entire muscle length. Human myosin heavy chain expression was further confirmed by Taqman real-time PCR using human specific primers (details below).

### Immunocytochemistry and immunohistochemistry

Heterogeneous cells and clonal progenies were incubated with the following primary antibodies for immunofluorescence: mouse monoclonal anti-CD-34 (sc-65261; Santa Cruz Biotechnology), CD105 (ab11414; Abcam), anti-CD146 (ab11372; Abcam), anti-flk1 (sc-57135; Santa Cruz Biotechnology), anti-Stro1 (MAB4315; Millipore), anti-human nuclei (HuNu, MAB1281; Millipore), anti-human dystrophin (dys, MAB1690, Millipore); rabbit polyclonal anti-CD44 (ab-51037; Abcam), anti-CD117 (ab16832; Abcam), anti-Oct4 (ab19857; Abcam), anti-Nanog (ab21603; Abcam), anti-MHC (MHC, sc-20641; Santa Cruz Biotechnology). Cells were washed in PBS, fixed with 10% formalin and permeabilized with 0.1% Triton X-100, and then blocked with buffer (LI-COR Biosciences). After incubation with primary antibodies, cells were washed and incubated with Alexa Fluor® 546 secondary antibodies (1∶200, Invitrogen) for CD117, Oct4, Nanog and MHC; Alexa Fluor® 488 nm and 594 nm secondary antibodies (1∶200, Invitrogen) for CD106, CD146, flk1, human nuclei and human dystrophin staining. Stro1 was detected by goat anti-mouse IgM-FITC (1∶200, sc-2859; Santa Cruz Biotechnology). Nuclei were visualized by DAPI staining. Muscle sections were air-dried and immunostained against human dystrophin, human nuclei and human PECAM [12].

### Real-Time RT-PCR Analysis

Real-Time RT-PCR was performed by extracting RNA from culture cells and muscle tissue. Total RNA was isolated using Trizol (Invitrogen) per manufacturer's protocols. RNA concentration was measured using UV spectrometer. A total of 1-µg RNA was used to synthesize cDNA (iScript™cDNA Synthesis Kit, BIO-RAD, Hercules, CA). The primer sets for real-time PCR assays were pre-designed TaqMan® human myosin heavy chain and human PECAM primers labeled with Fam (Applied Biosystems, Foster City, CA). GAPDH was used as housekeeping gene. The expression of candidate genes was determined with TaqMan® probe-based Universal PCR Master Mix (Applied Biosystems) using 7300 Real-Time PCR System (Applied Biosystems). The conditions for real-time PCR were as follows: 50°C, 2 min; 95°C,10 min; followed by 40 amplification cycles (95°C,15 s; 60°C, 60 s). Specific gene expression in each sample was calculated as ΔΔCt value.

### Statistical analysis

Power analysis was used to calculate sample sizes for *in vivo* experiments. For normal distribution, quantitative data of control and treated groups were subjected to One-way ANOVA and post-hoc Bonferroni tests. For skewed data, non-parametric Kruskal-Wallis tests were used.

## Results

### Stemness markers and population doubling

**Figure 1:**
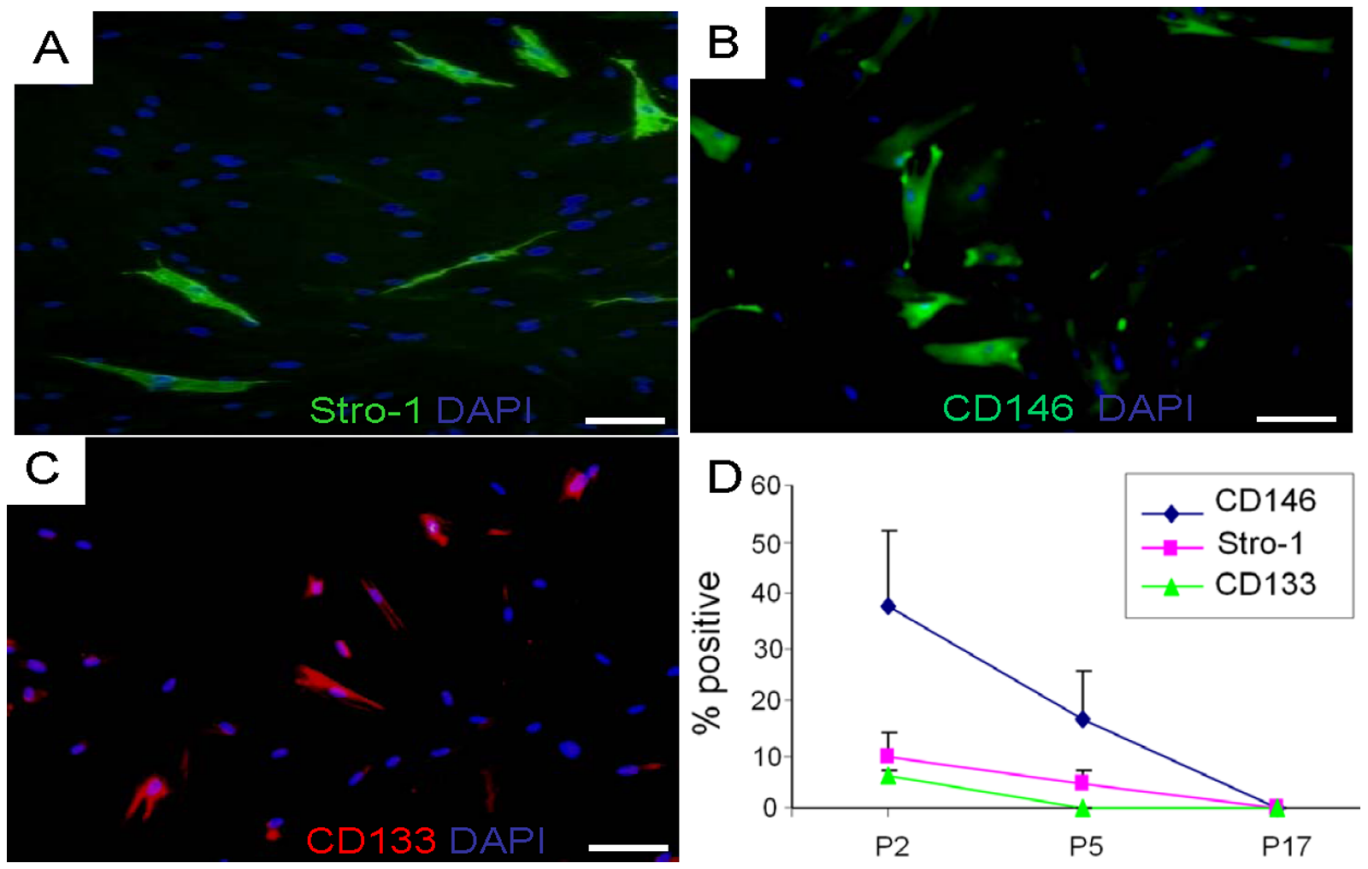
Characterization of heterogeneous stem/progenitor cells of the dental pulp. Immunostaining at passage 2 for Stro1(green) (A), CD146 (green) (B) and CD133 (red) (C) over DAPI-stained nuclei. Scale bars: 100 µm. D: Quantitative expression of CD146, Stro1 and CD133 at passages 2, 5 and 17 (N = 3).

**Figure 2:**
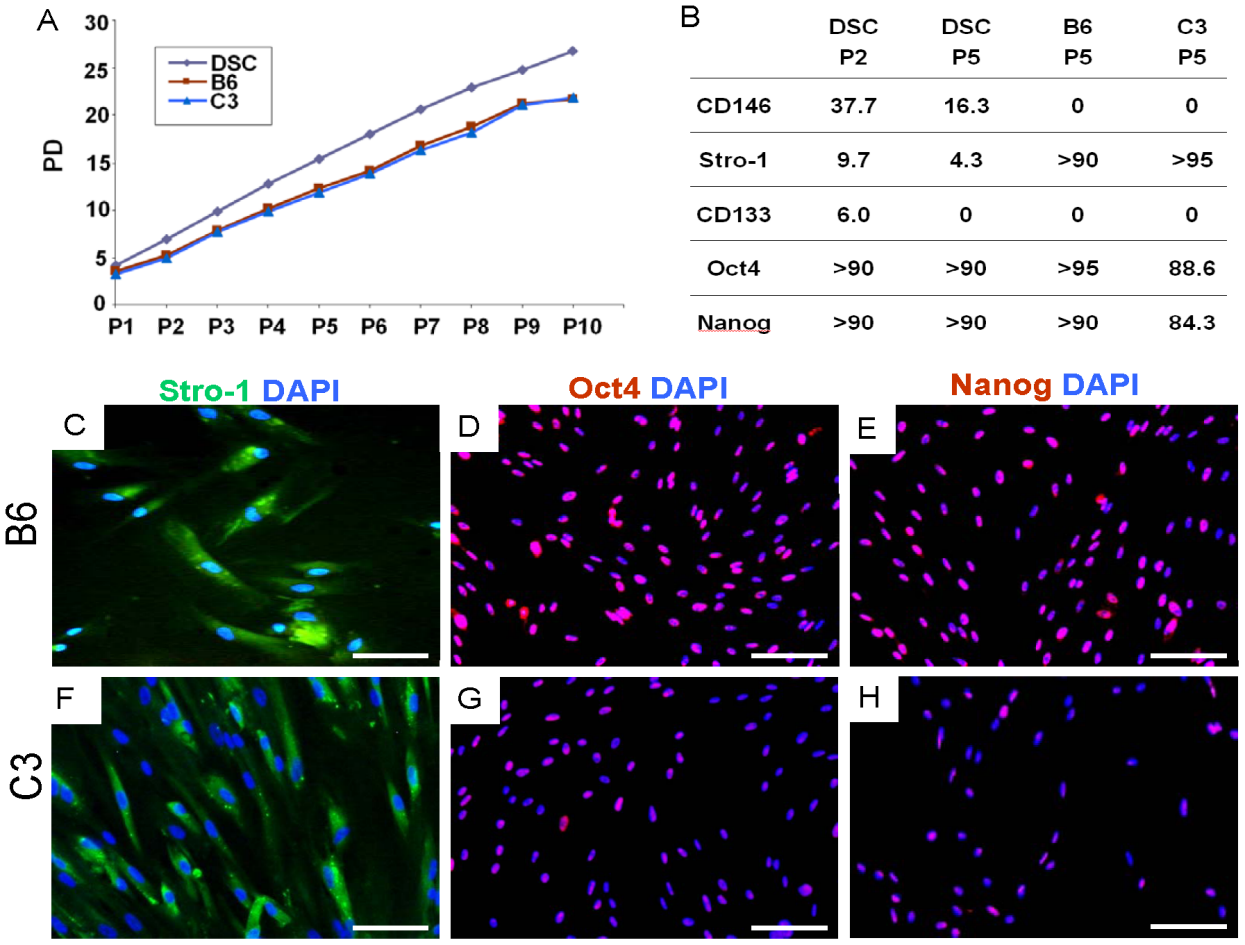
Myogenic clones in comparison with their parent stem/progenitor cells. A: Cumulative population doubling (PD) of two myogenic clones and their parent stem/progenitor cells showing a lack of statistically significant differences in PD kinetics. B: Expression of CD146, Stro1, CD133, Oct4 and Nanog by the two tested myogenic clones, B6 and C3, as well as their parent stem/progenitor cells. P: cell passage. Strikingly, B6 and C3 were overwhelmingly Oct4+, Nanog+ and Stro1+, and sustained the expression of these markers up to the tested 5 passages. C–H: Representative immunofluorescence of Stro1, Oct4 and Nanog by the two identified myogenic clones (passage 5) merged with DAPI-stain nuclei. Scale bar: 100 µm.

Passage 2 (P2), heterogeneous DSCs expressed Stro1, CD146 and CD133 (**Fig. 1A–C**), but did not express CD34, CD44, CD105 or flk1 (data not shown). However, the number of Stro1, CD146 and CD133 positive cells decreased with passaging. At P5, few CD133+ cells remained, whereas Stro1+ and CD146+ cells decreased substantially (**Fig. 1D**). By P17, Stro1, CD133 and CD146 were virtually absent in heterogeneous cells (**Fig. 1D**). Heterogeneous DSCs continued to undergo population doubling (PD) during the tested 10 passages (**Fig. 2A**). The two isolated myogenic clones, B6 and C3, showed similar morphology and PD kinetics to their parent cell population (**Fig. 2A**) (without statistically significant differences in PD), indicating that the isolated clones by limiting dilution from heterogeneous cell populations were as capable of self renewal as their parent mononucleated and adherent cell populations.

Out of the established 42 clones, cells from a total of 3 clonal progenies fused into multi-nucleated, MHC+ myotubes, including B6 and C3 (see below). The B6 and C3 clones were continuously passaged every 3–4 days over the tested 10 passages without apparent compromise in their doubling rate (**Fig. 2A**). Quantitatively, the percentage of heterogeneous cells that expressed CD146, Stro1 and CD133 were 37.7%, 9.7% and 6.0% at P2, but decreased to 16.3%, 4.3% and 0% by P5 (**Fig. 2B**). Strikingly, B6 and C3 clones were overwhelmingly Oct4+, Nanog+ and Stro1+, and sustained the expression of these markers up to the tested 5 passages (**Fig. 2B**; **Fig. 2C–E** **for B6**; **Fig. 2F–H** **for C3**). Contrastingly, B6 and C3 failed to express CD133 and CD146 (**Fig. 2B**). There were minimal differences between B6 and C3 clones in their morphology, population doubling rates, molecular marker expression or differentiation potential.

### Clonal progeny differentiated into myotube-like structures

**Figure 3:**
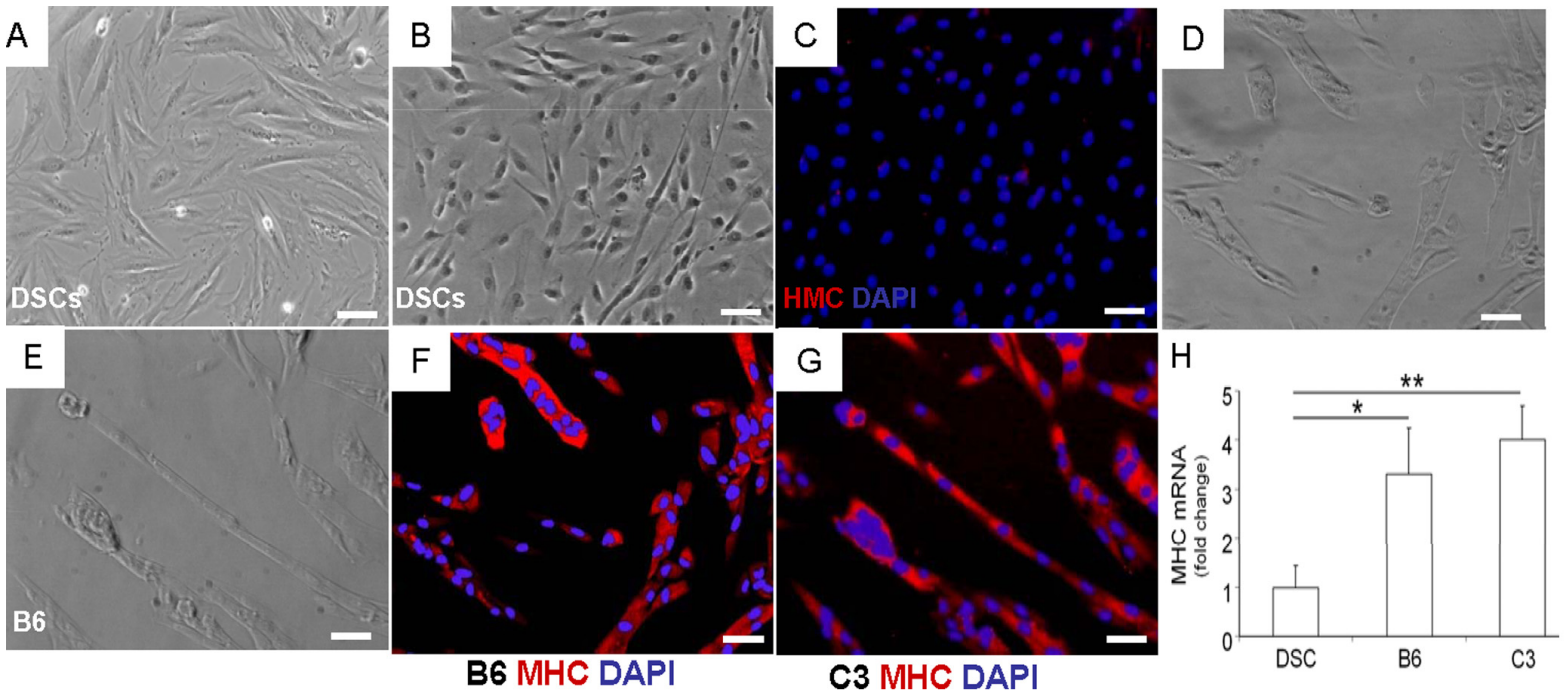
Myogenic potential of clonal progeny of ectopic dental stem cells in chemically defined medium. A: Phase contrast image of a representative heterogeneous dental stem cell (DSC) population showing typical spindle-shaped cells. Upon exposure to chemically defined medium, DSCs assumed spherical shape but failed to fuse into multinucleated cells (B) or express myosin heavy chain (C). Strikingly, two representative clones (B6 and C3) in chemically defined medium for 4 wks readily fused into tubular structures (D,E) that are positive for myosin heavy chain (MHC) immunoblotting (F,G). Quantitatively, MHC mRNA expression is significantly greater by B6 and C3 cells than their heterogeneous parent cells (DSC) (H). The y axis represents fold change related to heterogeneous DSC. Scale bar: 100 µm.

In contrast to their typical spindle shape (**Fig. 3A**), mononucleated and adherent cells of the tooth pulp upon 4-wk exposure to chemically defined myogenic differentiation medium assumed a somewhat rounded morphology (**Fig. 3B**). However, these heterogeneous stem/progenitor cells failed to fuse into multinucleated cells or express MHC (**Fig. 3C**). Strikingly, B6 and C3 exposed for 4 wks in the same chemically defined medium readily fused into multi-nucleated myotube-like structures (**Fig. 3D,E**) and robustly expressed MHC (**Fig. 3F,G**). Remarkably, fusion into multi-nucleated, MHC+ cells occurred in 4 wks without co-culture with mouse skeletal myoblasts (C2C12). Overall, ∼27% of B6 and ∼30% of C3 cells fused into multi-nucleated, MHC+ myotube-like structures that were quantified from multiple plates. Quantitative real-time PCR analysis revealed significantly greater MHC expression by B6 and C3 cells heterogeneous DSCs, from which B6 and C3 were cloned (**Fig. 3H**).

**Figure 4:**
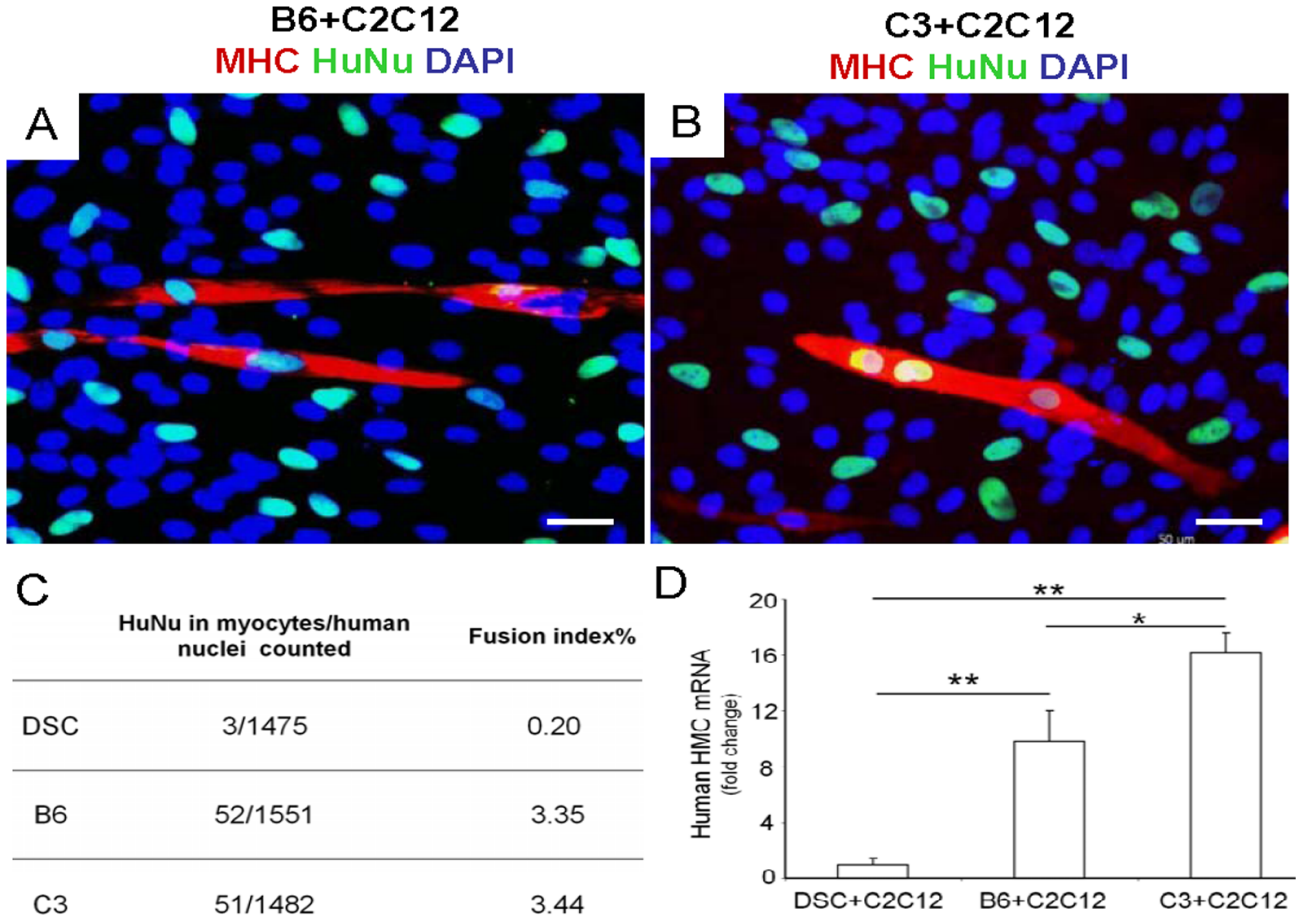
Myogenic potential of clonal progeny of ectopic dental stem cells upon co-culture with mouse skeletal myoblasts. Upon co-culture with mouse myoblast cell line (C2C12 cells) for 1 wk, some of the B6 and C3 cells again fused into multinucleated cells (A,B). Human nuclear staining indicates that the presence of human nuclei in some of the fused myotube like, MHC positive structures (A,B). Quantitatively, human nuclear fusion index (C) and human MHC mRNA expression of B6 and C3 (D) are significantly greater than DSC+C2C12 group. In (D), y axis represents fold change related to DSC+C2C12 group. Scale bar, 50 µm.

Upon co-culture with C2C12 cells, B6 and C3 also fused into multinucleated cells that expressed MHC within 1 wk (**Fig. 4A,B**). Human nuclear staining confirmed that multi-nucleated myotube structures contained both human and mouse nuclei (**Fig. 4A,B**). Upon co-culture of heterogeneous DSCs, 3 human cells (with positive human nuclear staining, HuNu) were identified among 1,475 cells, yielding a fusion index of 0.2% (**Fig. 4C**). In comparison, 52 human cells (with positive human nuclear staining, HuNu) were identified out of 1,551 cells when B6 clones were co-cultured with C2C12 cells, yielding a fusion index of 3.35% (**Fig. 4C**). Thus, the fusion index of myogenic clones was approximately 16–17 folds greater than their parent, heterogeneous stem cells. Furthermore, 51 human cells (with positive human nuclear staining, HuNu) were identified out of 1,482 cells when C3 clones were co-cultured with C2C12 cells, yielding a fusion index of 3.44% (**Fig. 4C**). Quantitative PCR revealed that co-culture of either B6 or C3 with C2C12 yielded significantly more human myosin heavy chain (MHC) mRNA than co-culture of heterogeneous DSCs with C2C12 (**Fig. 4D**).

### Myogenic clones engrafted into cardiotoxin paralyzed muscles *in vivo*

**Figure 5:**
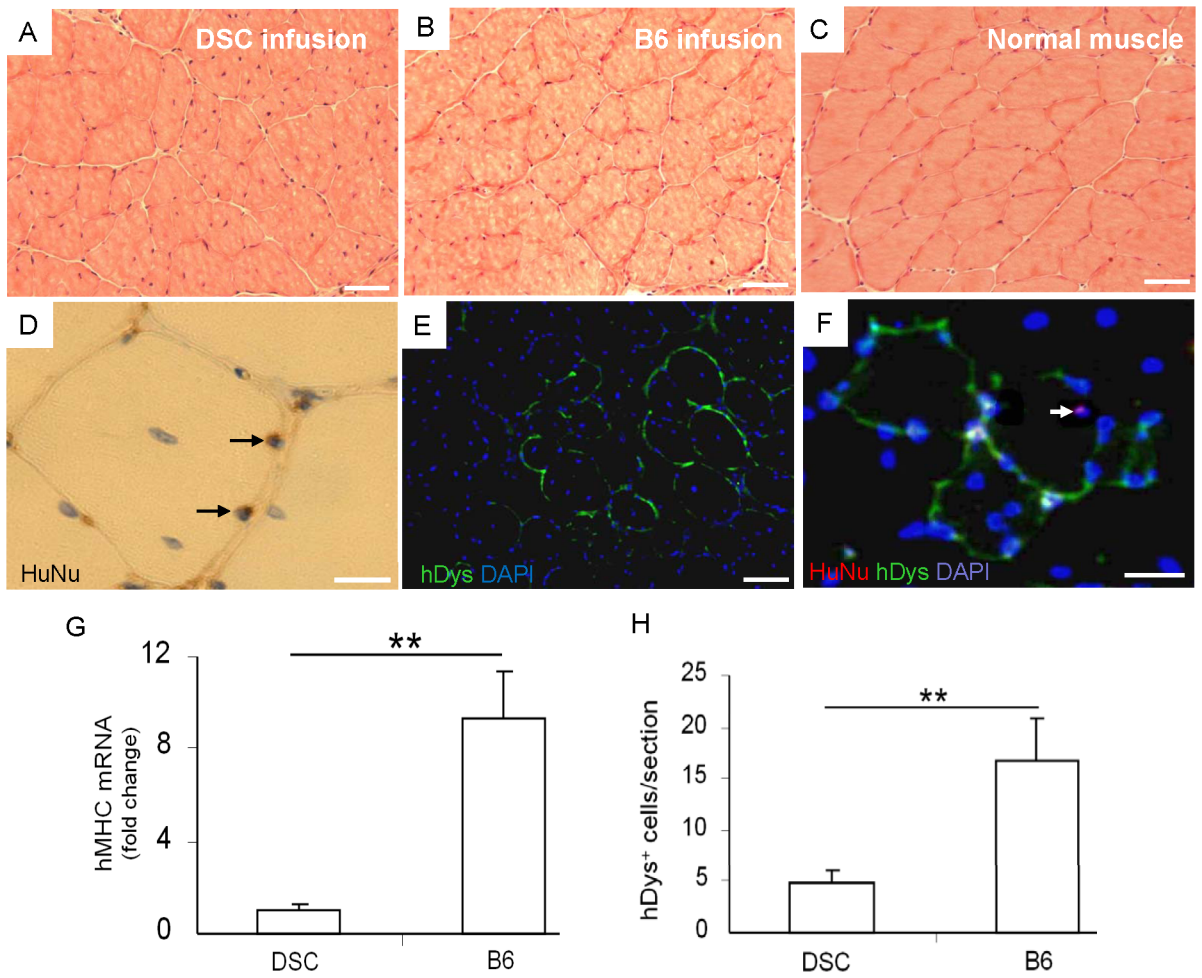
Engraftment of undifferentiated ectopic stem cell clone in damaged muscle. The tibialis anterior (TA) muscles in NOD/SCID mice were injured by multi-point cardiotoxin (CTX) injection. 24 hrs following CTX injections, dental stem cells (DSC) and a tested clone (B6) were separately infused in CTX-injured TA muscles with contralateral TA muscles as controls. A: H&E staining shows the presence of centralized nuclei in the representative DSC infused sample. B: The representative B6 infused sample showed abundant centralized nuclei. In contrast, the representative normal TA muscle has peripheral nuclei (C). Immunohistochemistry staining (brown) of human specific nuclei (D) and immunefluorescent staining of human specific dystrophin (green) and human nuclei (red) (E,F) indicates the presence of transplanted human cells in host TA muscle in the representative B6 infused group. We then harvested *in vivo* muscle samples, isolated RNA for real-time PCR analysis of myogenic differentiation *in vivo*. Quantitative RT-PCR assay revealed that human MHC gene expression in B6 infusion group after 4 wk injection is ∼8 times greater than DSC infusion group (N = 3, **p<0.01) (G). Quantification of human dystrophin positive cells present in the tibialis anterior (TA) muscle shows that the expression of human dystrophin mRNA was ∼3 times greater following B6 infusion than DSC infusion (N = 3, **p<0.01) (H). In (D) and (F), the arrows indicate the human nuclei. In G, y axis represents fold change relative to heterogeneous DSC. Scale bars: A–C, E, F: 50 µm; D: 20 µm.

The tibialis anterior (TA) muscles of NOD/SCID mice (N = 10) were injured by cardiotoxin (CTX) injection. Twenty-four hrs following CTX injection, B6 cells (at P5) and their parent, heterogeneous cells (both at 1×106 cells/mL) were infused separately into the injured TA muscles (N = 4 for B6; N = 6 for heterogeneous cells). CTX injured, PBS-injected and uninjured TA muscles without cell infusion served as controls. In comparison to peripherally located nuclei in the representative normal TA muscle specimen (**Fig. 5C**), abundant centralized nuclei were present upon infusion with heterogeneous cells or B6 clones (**Fig. 5A and 5B**), suggesting neomyofiber formation and muscle regeneration. Human nuclear staining confirmed the presence of engrafted (human) B6 cells (arrows in **Fig. 5D,F**). B6 infused cells showed substantial human dystrophin expression (**Fig. 5E,F**). Strikingly, B6-infused TA muscles expressed ∼9 times more human myosin heavy chain mRNA than TA muscles infused with their parent, heterogeneous DSCs (**Fig. 5G**) (p = 0.002). Additionally, the number of human dystrophin positive cells per section of B6-infused TA muscles was more than 3 times greater than that infused with heterogeneous DSCs from which B6 was cloned (**Fig. 5H**) (p = 0.008).

**Figure 6:**
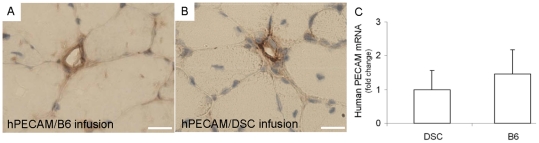
Contribution of transplanted myogenic clones and their parent stem/progenitor cells to angiogenesis. Immunolocalization of human-specific PECAM to a representative blood vessel (A) showing human cell derived endothelium upon infusion of a myogenic clone (B6). Similarly, infusion of heterogeneous stem/progenitor cells (DSC) also yielded human specific PECAM-positive blood vessel (B). Scale: 20 µm. C: Quantitative real-time PCR analysis showed a lack of significant differences in mRNA expression of human-specific PECAM by B6 and DSCs (p>0.05). Y axis represents fold change relative to heterogeneous DSCs.

Infused B6 and DSCs also formed chimeric blood vessels together with host endothelial cells. Some of the host blood vessel structures among muscle fibers showed positive human cell immunostaining with human-specific PECAM (**Fig. 6A,B**). However, human PECAM expression upon B6 infusion failed to show statistical advantage over human PECAM expression upon the heterogeneous DSCs infusion (**Fig. 6C**) upon quantitative real-time PCR analysis.

## Discussion

We discovered that clonal progenies of ectopic stem cells with robust *in vitro* myogenic capacity not only engraft in injured muscle, but also yield dystrophin and myosin heavy chain. Myogenic clones from ectopic stem cells, in this case dental stem cells, that natively do not differentiate into skeletal muscle, are more efficacious towards myogenic differentiation *in vivo* than their heterogeneous parent stem/progenitor cell populations. In comparison with the majority of previous approaches of muscle regeneration with heterogeneous stem/progenitor cells, the present findings demonstrate the potential value of myogenic clones out of ectopic heterogeneous cells for muscle repair. Some of the previous approaches for muscle repair have relied primarily on heterogeneous stem/progenitor cell populations including either muscle-derived or non-muscle stem cells [13]–[16]. Myogenic clones that are infused in muscle defects appear to generate high yield muscle cells *in vivo*, as shown by self-renewal and differentiation by a single muscle stem cell [9] and the expression of human dystrophin and MHC in the present work. Given the apparent biological advantage of myogenic clones, what are the issues for their potential application as a therapeutic cell source? The current view is that myogenic clones are scarce among ectopic stem cell populations. However, recent demonstration of robust self-renewal and differentiation capacity of a single muscle stem cell [9] provides grounds for exploring whether myogenic clones are capable of muscle healing. It is also possible, and yet untested, that muscle-derived stem cells or satellite cells yield more numerous myogenic clones than ectopic stem cells. Thus, cell therapies for muscle repair are likely effective by taking advantage of myogenic clones of both muscle-derived and ectopic stem cells. Dental stem cells were used as a model for ectopic stem cells in the present work because 1) DSCs can be readily isolated from extracted teeth that are currently treated as medical waste, and thus have great potential for cell therapies, and 2) DSCs natively do not differentiate into muscle cells and therefore qualify as ectopic stem cells for myogenic differentiation.

One of the striking findings in the present study is that ectopic stem cells isolated from dental pulp fail to fuse into multinucleated myofiber-like structures despite exposure to chemically defined medium that is known to induce myogenic fusion of several other postnatal stem cell populations such as bone marrow or adipose [4], [17]. The mechanisms of cell fusion are not well understood, although several cellular.

machineries including cell membrane proteins and associated signaling appear to be highly conserved for cell fusion and differentiation into myocytes [18]–[19]. Few myogenic stem/progenitor cells are present among heterogeneous stem cells of dental pulp in the present study: only 3 out of 42 clones showing myogenic potential. It is conceivable that the scarce myogenic cells fail to fuse with adjacent cells most of which are not myogenic and do not express the same cell surface proteins that are putatively important for cell fusion. Conversely, myogenic clones in the present study, B6 and C3, readily fuse even without co-culture with mouse myoblast cell line, C2C12 cells which are known to fuse with other myocyte-like cells.

We found a lack of statistically significant differences in mRNA expression of human PECAM by the transplanted heterogeneous DSCs and their clonal progeny cells. This appears to suggest that while myogenic clones of ectopic stem cells show advantage in myogenic capacity, they may not necessarily elaborate more vasculature. Previous work has shown that transplanted stem/progenitor cells are capable of co-endothlializing blood vessels with host cells [20]–[21], [12], [22]. Whereas the isolated myogenic clones are capable of engrafting with host muscle fibers and yield myosin heavy chain without necessarily relying on enhanced angiogenesis. Coupled with the paucity or decreases in the expression of CD146, an endothelial progenitor marker by either heterogeneous DSCs or their myogenic clones, it is somewhat surprising that the derived myogenic clones appear to be capable of muscle repair without necessarily an accompanied enhancement in angiogenesis. However, much additional work is warranted to ascertain the roles of angiogenesis in muscle regeneration by clones of ectopic stem/progenitor cells. The myogenic clones in the present work are sustainably Oct4+, Nanog+ and Stro1+, but CD133- and CD146-. As parent heterogeneous cells deplete their expression of Stro1, CD133 and CD146, the myogenic clonal progenies maintained robust expression of Stro1, Oct4 and Nanog. This appears to be consistent with weak CD133 and CD146 expression by human pericytes in muscle regeneration [7]. Much meritorious effort has been directed towards transcriptional control of myogenic differentiation from stem/progenitor cells. The present data represent a rare glimpse of *in vivo* muscle repair by clonal progenies of heterogeneous stem/progenitor cells with robust capacity for myogenic differentiation *in vitro*.
